# ADHD and its neurocognitive substrates: A two sample Mendelian randomization study

**DOI:** 10.1038/s41398-022-02139-x

**Published:** 2022-09-09

**Authors:** Kwangmi Ahn, Luke J. Norman, Cristina M. Justice, Philip Shaw

**Affiliations:** 1 Neurobehavioral Clinical Research Section, Social and Behavioral Research Branch, National Human Genome Research Institute, National Institutes of Health, Bethesda, MD 20892 USA; 2 National Institute of Mental Health, Bethesda, MD 20892 USA

**Keywords:** Comparative genomics, ADHD

## Abstract

Attention-deficit/hyperactivity disorder (ADHD) is associated with a wide array of neural and cognitive features, and other psychiatric disorders, identified mainly through cross-sectional associations studies. However, it is unclear if the disorder is causally associated with these neurocognitive features. Here, we applied a two-sample bidirectional Mendelian randomization (MR) study to summary GWAS data to explore the presence and direction of a causal effect between ADHD and a range of neurocognitive features and other psychiatric disorders. The inverse variance weighted method was used in the main analysis, and two MR methods (MR-Egger, weighted median) were used for robustness checks. We found that genetic risk for ADHD was causally associated with a decreased area of lateral orbitofrontal cortex. Conversely, we found that brain volume and some features of intrinsic functional connectivity had causal effects on ADHD risk. Bidirectional causal links were found between ADHD and adult general intelligence, as well as depression and autistic spectrum disorders. Such work highlights the important ties between ADHD and general cognitive ability, and suggest some neural features, previously merely associated with the disorder, may play a causal role in its pathogenesis.

## Introduction

Attention deficit hyperactivity disorder (ADHD) is a highly heritable neuropsychiatric disorder, with family and twin studies suggesting a heritability of around 70–80% [1]. A landmark study from the Psychiatric Genetics Consortium has identified 27 genome-wide significant common variants to be associated with ADHD [2]. T Using exome sequence data, fine-mapping of these loci highlighted risk genes enriched among genes expressed in the embryonic frontal cortex. The ADHD risk genes also overlapped with genes expressed in dopaminergic midbrain neurons, long held to be important in the pathogenesis of the disorder. Overall, common variants in ADHD explained around 22% of the variance in the phenotype. Rare, structural genomic variants also play a role in the pathogenesis of the disorder, with meta-analyses finding an enrichment of CNVs among genes that show neural expression, and there is modest convergence between the genes implicated by CNVs and those implicated by GWAS [3].

Complimenting the genetic associations studies of ADHD, a wide range of associations have been reported between ADHD and cognitive and neural features. The disorder is strongly associated with other psychiatric disorders, particularly other neurodevelopmental disorders, such as autistic spectrum disorders (ASD) [4, 5] and Tourette’s syndrome (TS) [6]; along with mood and anxiety disorder (including major depression, bipolar affective disorder) [7]. However, a critical limitation of most studies is their cross-sectional design. While valuable, this study design can only indicate associations between ADHD and its putative neurocognitive substrates and causal relationships cannot be inferred. Similarly, while ADHD may be associated with other disorders such as ASD, it is unclear if the presence of one disorder causally increases the risk for another.

To overcome this limitation, an epidemiological approach has been proposed, Mendelian Randomization (MR), that uses genetic variants as instrumental variables to assess potential causal relationships between an exposure (such as liability to ADHD) and an outcome (such as neural features) [8]. Two-sample MR is an extension of MR that allows the instrument-exposure and instrument-outcome associations to be measured in two independent samples [9]. This technique increases power by allowing the inclusion of data from large GWAS consortia and is less influenced by potential confounding due to the random allocation of alleles at conception [10].

To conduct MR, genetic data, usually in the form of genome wide association study, is needed on both the “exposure” (e.g., ADHD) and the “outcome” (e.g., associated neural features). Such GWAS data exists for ADHD, stemming from Psychiatric Genomics Consortium ADHD working group, as does publicly accessible GWAS summary results for many traits and disorders associated with ADHD. The application of MR to such summary GWAS data has already allowed the exploration of possible causal pathways between ADHD and other mental and physical disorders. For example, the MR approach has demonstrated a possible causal effect of liability to ADHD on major depression [11], smoking, cannabis use, and, tentatively, alcohol dependence (but not from substance use to ADHD) [12]. ADHD has also been causally linked with physical conditions, including coronary artery disorders and obesity [13].

Here we extend this work by examining possible causal links between ADHD and an array of neural and cognitive features. In focusing on neural features, we leverage recent mega-analyses from the ENIGMA consortium, which report on the anatomic features that are most robustly associated with ADHD. The ENIGMA-ADHD consortium has reported modest associations between ADHD and total brain volume, and at the subcortical level, with striatal and amygdala volume reduction [14]. At the cortical level, the most significant ADHD-related differences were reductions in the area of the right lateral orbitofrontal and superior frontal cortex, and a thinner right fusiform and precentral cortex [15]. Fortunately, summary GWAS data on these neuroanatomic features is also available, from the UK Biobank, making it possible to include these ADHD-associated anatomic features in a MR study.

ADHD has also been hypothesized to be a disorder of neural dysconnectivity [16–18]. This is supported by a meta-analysis of anomalies in white matter microstructure [19] which find reductions in a marker of microstructure -fractional anisotropy- within the corpus callosum, (which provides inter-hemispheric communication), the right cingulum (encompassing tracts that integrate frontal, temporal and parietal cortical regions) and the right sagittal stratum (connecting subcortical striato-thalamic regions with the cerebellum). Anomalies of connectivity at a functional level, mainly parsed by resting-state functional MRI data, further support the dysconnectivity hypothesis and are often interpreted within default mode interference models of the disorder [20]. These models point to abnormalities involving the default mode network, which is putatively involved in off-task, nondirected cognition, and its interactions with the task-positive executive control and salience networks which support effortful, goal-driven cognition and externally focused attention [21]. Imbalances in the interplay between these networks has been evidenced by recent meta-analyses of cross-sectional resting-state functional connectivity studies comparing patients with ADHD and unaffected controls [22], and have been postulated to underlie the attention lapses and behavioral and cognitive variability that characterize many of the cognitive difficulties associated with the disorder [23–26]. Here we again leverage a recent meta-analysis, selecting the patterns of altered intrinsic connectivity that are most strongly associated with ADHD. These connectivity features also benefit from GWAS into their genetic associations, again allowing the use of MR to move beyond association towards parsing causal relationships.

While no single cognitive feature characterizes ADHD, small changes have been reported in more general measures of cognition, such as “g” (the first component extracted from a swathe of cognitive tests) or measures such as the intelligence quotient [27]. Additionally consistent differences are reported variability in the time taken to make responses, which is taken to represent the effects of momentary lapses in attentional focus [28–31]. Again, we leverage GWAS data on these cognitive measures to examine possible casual links with ADHD. Other cognitive differences reported in ADHD, such as working memory, reward processing and some facets of emotion processing, lack such GWAS data and so cannot be considered using the MR approach. Finally, while previous MR studies have examined the links between ADHD and depression, ASD, substance use disorders, self-harm [32], and neuroticism [33], we extend the search to links with schizophrenia, bipolar disorder, anxiety disorder, Tourette’s, and obsessive compulsive disorder.

## Materials and methods

### Data sources

We obtained summary GWAS data on ADHD from PGC on participants of European ancestry (the 11 PGC samples and the 23 genotyping batches within iPSYCH) [34]. The meta-analysis included results for 8,047,421 markers and 304 genetic variants in 12 independent loci that surpassed the threshold for genome-wide significance (*P* < 5 × 10^−8^). Summary GWAS data for each the neurocognitive traits and for other psychiatric disorders were obtained from publicly available datasets, as shown in Table 1.

**Table 1:** Phenotypes examined, the source of the summary GWAS data, and the sample size in each GWAS.

Phenotype	Data source	Sample size
ADHD	PGC (2019)	53,293
Neuroanatomic features		
Anatomic		
Total brain volume and related metrics	Jansen et al. (2020)	47,316
Left Amygdala volume		
Right Caudate volume		
Right Putamen volume	Smith et al. (2021)	33,224
Area of right lateral orbitofrontal cortex		
Area of right superiorfrontal cortex		
Thickness of right fusiform cortex		
Thickness of right precentral cortex		
White matter tract microstructure (fractional anisotropy)		
Right sagittal stratum	Smith et al. (2021)	33,224
Splenium of the corpus callosum		
Left tapetum		
Intrinsic functional connectivity		
Default mode/executive/salience (dorsolateral) component (ICA2)		
Default mode, salience/executive, attention networks (parietal) component (ICA3)	Zhao et al. (2020)	34,691
Default mode, salience/executive, attention networks (angular) component (ICA4)		
Default mode/executive/salience (inferolateral) component (ICA6)		
Cognition		
Measures of general cognitive ability		
Childhood cognitive ability	Benyamin et al. (2014)	12,441
Adult Intelligence (“g”)	Savage et al. (2018)	269,867
Attention/reaction time		
Reaction time in children	Alemany et al. (2016)	1665
Reaction time in adults	Davies et al. (2019)	330,069
Intra-individual response time variability: factor reflecting selective attention	Pinar et al. (2018)	857
Mental disorders		
Schizophrenia	PGC (2018)	35,802
Bipolar Disorder	PGC (2021)	413,466
Major Depression	PGC (2019)	480,359
Anxiety Disorder	PGC (2016)	17,310
Autism Spectrum Disorder	PGC (2019)	46,350
Tourette’s Syndrome	PGC (2019)	14,307
Obsessive Component Disorder	PGC (2018)	9725

### ADHD-associated traits

Details on trait definitions and the origin of the summary GWAS data are given in Supplementary Table 1.

### Brain phenotypes

We selected neuroanatomic features that have been associated with ADHD through recent mega and meta-analytic studies. This included brain volume, and the closely correlated measures of intracranial volume and head circumference. We also considered the subcortical features most robustly associated by mega-analyses with ADHD, namely the volume of the left amygdala (ADHD related change d = −0.19), right caudate (d = −0.11), and right putamen (d = −0.14) [35]. At a cortical level we included thickness of the right fusiform and right precentral grays; surface area of the right orbitofrontal (d = −0.17) and superior frontal gyrus (d = −0.19) [15].

White matter tract microstructural measures of the tight sagittal stratum, splenium of the corpus callosum and left tapetum were chosen based on the voxel wise meta-analysis of fractional anisotropic changes associated with ADHD [19].

For intrinsic functional connectivity, we leveraged a large GWAS of resting state connectivity data (*N* = 34, 691) [36]. This study applied spatial Independent Component Analysis to resting-state adjacency matrices, thus summarizing 1695 pairwise connections. Six global metrics were extracted and we retained the four significantly heritable metrics (h2 = 0.48 to 0.61). Each of the four components captures different connectivity patterns between regions of the default mode, executive control and salience networks (for ICA2 and ICA6) extending in some components to attention networks (ICA3 and ICA4). While the differences between the components are complex, ICA2 consistently involves regions of dorsolateral (superior and middle) prefrontal cortex connecting with different posterior brain regions (mainly in the DMN, central executive and salience networks) whereas ICA6 had its anterior brain node centered on inferior frontal regions. By contrast, ICA 3 and ICA4 both incorporate the attention networks, but ICA3 consistently centered on Supramarginal and inferior parietal cortical regions, whereas ICA4 consistently involves angular regions of the default mode network. For ease of nomenclature, we thus refer to ICA2 as the default mode/executive/salience (dorsolateral) component; and ICA6 as the default mode/executive/salience (inferolateral) component. ICA3 is labeled as the default mode, salience/executive, attention networks (parietal) and ICA4 as default mode, salience/executive, attention networks (angular). These brief descriptions are heuristics and not exhaustive descriptions of the complex components. At the highest level however, each component aligns with current concepts of ADHD as the endpoint of disrupted connectivity between the default mode and task positive networks.

### Cognitive traits

Cognitive traits were selected based both on associations with ADHD, and publicly available GWAS summary-level data on underlying SNP associations. These include childhood and adult general intelligence. The childhood study reported GWAS on both the intelligence quotient (IQ) and “g” (the first unrotated factor of a factor analysis of a set of cognitive tests; the adult GWAS focused on “g” (derived from a range of measures of intelligence in across multiple cohorts).

We also considered measures derived from the Attention Network Test (which had GWAS data on 1665 youth): namely attention alerting, orienting, executive attention, reaction time and standard error of reaction time. Reaction time was also considered, using GWAS data from the UK BioBank. Finally, we considered measures stemming a GWAS into intra-individual response time variability (IIRTV), as a putative measure that is tied to attentional brain network activity. The GWAS into IIRTV extracted these measures from two tasks, and derived principal components: the first component was taken to reflect response selection, as it loaded on response time in tasks that required participants to select one response from competing choices. The second component was taken to reflect selective attention as it comprised of response time tasks that necessitated participants to choose task-relevant from task-irrelevant stimuli.

### Other disorders

We included seven mental disorders: Schizophrenia, Bipolar Disorder, Major Depression, Anxiety Disorders, ASD, Tourette’s Syndrome and Obsessive Compulsive Disorder. All summery level GWAS data sets for mental health were from PGC (https://www.med.unc.edu/pgc/).

### Genetic instrumental variable (IV) selection

Prior to extracting genetic instruments for each exposure (trait), linkage disequilibrium (LD) clumping was applied to the summary GWAS data to select independent SNPs (r^2^ = 0.01, with a 10,000 kb window). Linkage disequilibrium among SNPs was calculated based on 1000 genomes LD reference panel (European population). We then extracted SNPs associated with each exposure at a genome-wide level of significance (*P* < 5 × 10^–8^). Finally, we harmonized exposure and outcome data so that the effect estimates of both exposure and outcome variants were expressed per effect allele increase. Palindromic SNPs, in which the alleles on the forward strand are the same as on the reverse strand (e.g., A/T on forward strand and T/A on reverse strand) were excluded. For traits that had 3 or fewer IVs at this level of significance level, we relaxed the P-value threshold to *P* < 1 × 10^−5^. This relaxed threshold was used for childhood cognitive ability, attention function (reaction time) in children, the component reflecting selective attention derived from intra-individual response time variability measures, anxiety disorders, ASD, and Obsessive Compulsive Disorder. Traits with three or fewer independent SNPs (associated at the *P* < 1 × 10^−5^) were excluded for the further analysis. Dropped traits due to the lack of IVs are listed in Supplementary Table 1. *P*-value threshold and number of IVs for each MR analysis are included in Supplementary Table 2.

### Mendelian randomization analysis

We conducted bidirectional two-sample MR analyses to test for the causal relationship between risk ADHD and related traits. We used the inverse the variance–weighted (IVW) method [37] as the primary analysis. Because IVW method gives an unbiased estimate only if IV are valid or no pleiotropy [37], we conducted sensitivity analyses to assess the pleiotropy or outliers before running the IVW method. First, we estimated the intercept from MR-Egger Regression [38]. An intercept from MR-Egger significantly different to zero (*P* < 0.05) is an indication of directional pleiotropy (horizontal pleiotropy). Secondly, we used pleiotropy residual sum and outlier (MR-PRESSO) test [39] to identify horizontal pleiotropic outliers. If detected, we removed these outliers from the analyses. Thirdly, we assessed causality using weighted-median regression, which gives unbiased estimates provided at least 50% of the information comes from non-pleiotropic SNPs. Finally, we performed leave-one-out analysis to test if the result was being driven by any one variant.

### Power analysis

We calculated the power of our MR analyses using the online calculator mRnd (https://shiny.cnsgenomics.com/mRnd/) [40]. The equations use an approximate linear model on the observed binary (0–1) scale, and requires a proportion of the variance in the exposure variable be explained by SNPs, true odds ratio of the outcome variable per standard deviation of the exposure variable and the sample size.

All statistical analyses were performed using the TwoSampleMR [41] and MR-PRESSO [39] packages in R Software 4.1.0. To correct for multiple testing, we applied Benjamini-Hochberg’s False Discovery Rate (FDR < 0.05).

## Results

We applied two sample bidirectional MR to parse causal relationships between genetic risk for ADHD and 27 ADHD associated traits. Statistical power is given Supplementary Table 3. There was good power for MR examining links from ADHD to ASD, and moderate power for detecting links between ADHD and both depression and anxiety disorders. We were similarly well-powered to detect links from ASD, major depression, and schizophrenia to ADHD.

### Bidrectional links

**Fig. 1:**
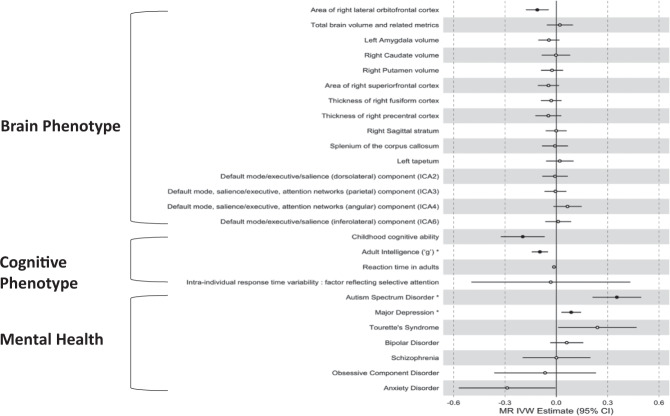
Casual Effect of ADHD to related traits (ADHD → Related traits). Closed circle FDR-adjusted *P*_IVW_ < 0.05. *Bidirectional casual associations were found.

**Fig. 2:**
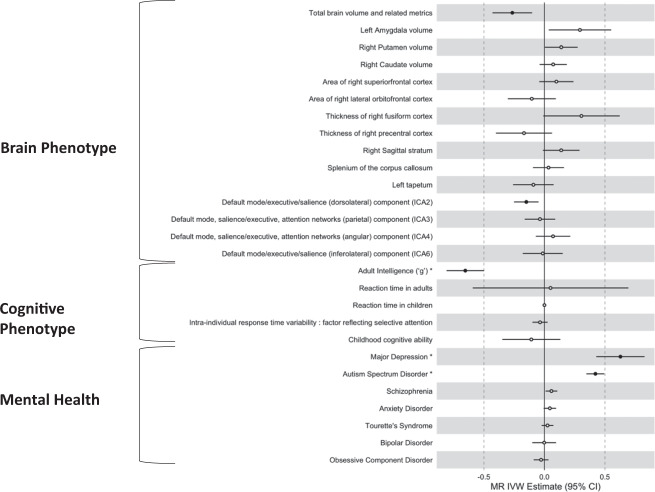
Casual Effects of related traits on ADHD (Related Traits → ADHD). Closed circle FDR-adjusted *P*_IVW_ < 0.05. *Bidirectional casual associations were found.

Bidirectional casual associations were detected between ADHD and intelligence. Firstly, genetic variants associated with ADHD were causally associated with “g”, the measure of adult intelligence (*ß*_IVW_ = −0.096; 95% CI: −0.144 to −0.048; FDR-adjusted *P*_IVW_ = 0.001). (Fig. 1) Conversely, 180 independent SNPs associated with adult “g” were causally associated with the risk of ADHD: higher intelligence was associated with a 48% reduction in of ADHD (OR = 0.52; 95% CI: 0.45–0.61; FDR-adjusted *P*_IVW_ = 1.75 × 10^−15^). (Fig. 2) The relationship between ADHD and childhood intelligence was unidirectional, with liability to ADHD being casually linked with intelligence, but not vice versa (*ß*_IVW_ = −0.196; 95% CI: −0.324 to −0.068; FDR-adjusted *P*_IVW_ = 0.016).

A bidirectional causal association was also detected between major depression and ADHD: liability to ADHD increased the risk of MDD by ~9% (OR, 1.09; 95% CI: 1.03–1.15; FDR-adjusted *P*_IVW_ = 0.016) and liability to major depression increased the risk of ADHD by ~87% (OR = 1.87, 95% CI: 1.53–2.29, FDR-adjusted *P*_IVW_ = 6.62 × 10^−9^). Liability to ADHD was also associated with an increased risk of ASD (OR, 1.42; 95% CI: 1.23–1.64; FDR-adjusted *P*_IVW_ = 3.19 × 10^−5^). The converse directional link of liability to ASD increasing risk for ADHD emerged only at the threshold of *P* < 1 × 10^−5^, (encompassing 50 SNPs), (OR = 1.52; 95% CI: 1.42–1.64; FDR-adjusted *P*_IVW_ = 4.55 × 10^−28^).

### Traits causally associated with risk for ADHD

We found that two neural traits were associated with risk for ADHD. Firstly, IVW using 16 SNPs associated with total brain volume showed greater brain volume was associated with lower risk of ADHD (*ß*_IVW_ = −0.265; 95% CI: −0.429 to −0.102; FDR-adjusted *P*_IVW_ = 0.01) (Fig. 2).

A causal effect also emerged for one of the global resting-state connectivity components and ADHD, namely the component with an anterior dorsolateral prefrontal cortical node that connected to a range of posterior regions in the default mode, central executive and salience networks. Thirty SNPs associated with this connectivity component were causally associated with the risk of ADHD. The IVW results showed that greater expression of this component during rest was associated with a lower risk of ADHD (OR = 0.86; 95% CI: 0.78–0.95; FDR-adjusted *P*_IVW_ = 0.02). No white matter microstructural measures showed possible casual effects.

### Traits causally linked to ADHD

A unidirectional link between liability to ADHD and one neuroanatomic trait: area of the right lateral orbitofrontal cortex. Specifically, ADHD was casually linked to a decrease in the area of the right lateral orbitofrontal cortex (*ß*_IVW_ = −0.111; 95% CI: −0.178 to −0.044; FDR-adjusted *P*_IVW_ = 0.01). ADHD was not linked casually to any other anatomic, white matter microstructural and intrinsic functional connectivity.

As mentioned above, we also found an unidirectional link between liability to ADHD risk and childhood cognitive ability (*ß*_IVW_ = −0.196; 95% CI: −0.324 to −0.068; FDR-adjusted *P*_IVW_ = 0.016).

### Sensitivity analysis

Using the final SNP set for each analysis, we found no evidence of horizontal pleiotropy by MR-Egger, and no outliers detected by MR-PRESSO (Supplementary Table 4). Leave-one-out analysis also showed that the results were not influenced by a single outlying variant (Supplementary Figs. 1–10). The effect estimates were directionally consistent across the sensitivity analyses performed (Supplementary Table 5).

## Discussion

In this study, we applied two sample MR analyses to investigate the magnitude and direction of causality between ADHD with its related traits. We found that liability to ADHD was causally linked with a decreased area of the right lateral orbitofrontal cortex. Conversely, we found that brain volume and features of global functional connectivity had causal effects on ADHD risk. Bidirectional causal links were found between ADHD and measures of adult (but not childhood) intelligence, depression, and autistic spectrum disorder.

The most novel findings pertain to the possible causal relationship between neural features and ADHD. We found that decreased expression of a global functional connectivity component, centered on dorsolateral prefrontal cortex and encompassing connections between regions of the executive control, default mode and salience network, had a causal association with increased risk for ADHD. The executive control, default mode and salience network are closely implicated in ADHD and previous work has pointed to cross-sectional associations between ADHD symptoms or diagnosis and abnormal intra- and inter-network connectivity involving these network [23–26]. While an implicit assumption in much of the existing neuroimaging literature on ADHD has been that these functional brain abnormalities play a causal role in the onset of ADHD symptoms, this has rarely been studied directly in previous work [42]. The use of a Mendelian randomization design therefore provides important genetically informed evidence for a causal relationship between tripartite network connectivity and ADHD diagnosis. Such findings are important in light of ongoing efforts to develop biomarker-driven interventions for the disorder, which require a delineation of functional brain abnormalities that play etiological roles in the onset of ADHD from those that are secondary consequences or merely epiphenomenal correlates of the disorder [42, 43].

Structurally, we found that total brain volume had a causal relationship with risk for ADHD, such that smaller brain volumes were positively associated with ADHD risk. Smaller brain volumes, involving smaller gray matter and white matter volumes, have been reported often in pediatric ADHD samples. Specifically, patients with the disorder have been reported to show approximately 1–5% decreases in total cerebral volume, including total white and grey matter volume, compared to matched controls [44–50]. Of note, reduced overall cortical thickness, cortical surface area and cortical folding have been also reported in patients with ADHD [51, 52]. These observations have been supported mega-analytically, with findings from the multi-site ADHD-ENIGMA study pointing to widespread decreases in gray matter volume, thickness and surface area (*d* = 0.1 to *d* = 0.21), including reduced overall intracranial volume (2%; *d* = 0.14) in children with ADHD relative to controls. Previous work has indicated relationships between reduced total brain volume and genetic risk for ADHD, both as assessed using polygenic risk scores [53], and in studies of “at risk” unaffected siblings [48]. Specifically, in the latter study, probands with ADHD were reported to show reductions in total brain volume of around 3% relative to healthy controls (*N* = 196), while their unaffected siblings (*N* = 169) showed total brain and total gray matter volumes that were intermediate to participants with ADHD and control individuals [48]. Our finding, in conjunction with this existing work, provides converging evidence for a mechanistic role for reduced total brain volume in the onset of ADHD [48]. Importantly, ADHD was found to be a risk factor for reduced surface area of right lateral orbitofrontal cortex (residualized for total brain volume). This finding suggests that, in addition to a predominant causal chain from total brain volume to ADHD diagnosis, local alterations in brain structure may also occur as a downstream consequence of the disorder on brain development.

Both epidemiological and genetic studies suggesting that ADHD is associated with cognitive differences, and vice versa. For example, measures of childhood cognitive ability and verbal numeric reasoning show an inverse genetic correlation with ADHD (*r*_g_, −0.41, −0.36, respectively) [34]. A recent study found the bidirectional negative casual effects between ADHD and educational attainment, an outcome that is modestly associated with general intelligence [54]. We further report bidirectional causal links between ADHD and a more direct measure of adult intelligence.

We confirm prior findings of bidirectional causal effects between ADHD and major depression, using the same summary GWAS data, but slightly different clumping methods [11]. It might seem unusual that MDD, which usually has an adolescent or adult onset, can act as a risk factor for a childhood onset disorder such as ADHD. It is important to recall that MR focuses on the genetic risk for MDD, not the actual expression of the disorder, and it is already well established that there is positive genetic correlations between ADHD and MDD (reported ot be r_g_ = 0.42) [34, 55]. Our study also showed that ADHD increased the risk of ASD by 48%, and while an effect of ASD risk on ADHD did not emerge when SNPs attaining GWAS significance level were used as IVs, this relationship did emerged when nominally significant SNP were used, as has been previously reported [56].

There are limitations to the study. Firstly, we had limited power to detect causal effects for some traits. MR methods depend on the source GWAS samples sizes and heritability of the trait. These factors may have had a particular impact on the GWAS summary data sets for attention, and intra-individual response time variability, as all GWAS were based on modestly sized samples. Many cognitive traits associated with ADHD, such as reward processing, visuospatial working memory and sustained attention performance still lack the publicly available GWAS data needed for MR. Thirdly, despite applying a number of methods to test for pleiotropy and finding limited evidence for any directional pleiotropy, we cannot fully rule out possible bias due to residual horizontal pleiotropy.

In conclusion, we provide further evidence for a causal link between cognition and ADHD. We also point to ADHD as a possible driver of observed changes in the lateral orbitofrontal cortex, and to alterations in features of functional connectivity as a possible driver of the disorder.

## Supplementary information

Trait definitions and the origin of the summary GWAS data

P-value threshold and number of IVs for each MR analysis

Statistical power for MR analysis

Test of Outliers by MR-PRESSO

Leave one out Analysis

Sensitivity analyses
