# Supplementary material for: ADHD and its neurocognitive substrates: A two sample Mendelian randomization study
Source: Transl Psychiatry. 2022 Sep 9;12:378. doi: 10.1038/s41398-022-02139-x (PMC9463186; doi:10.1038/s41398-022-02139-x)

Supplementary Figure 1. Leave one out Analysis: ADHD 🡪 Area of Right latera lorbitofrontal


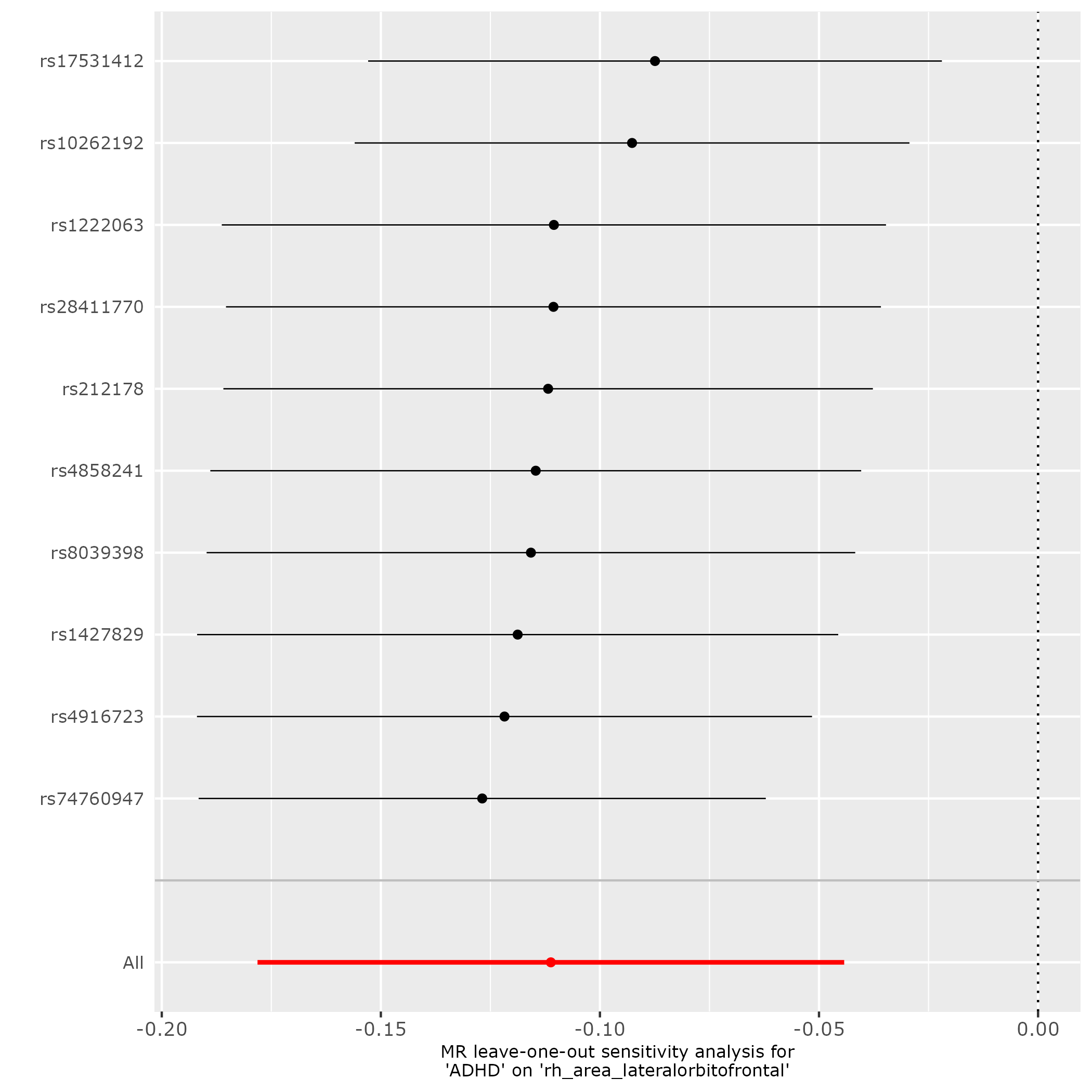


Supplementary Figure 2. Leave one out Analysis: ADHD🡪 Childhood Cognitive ability


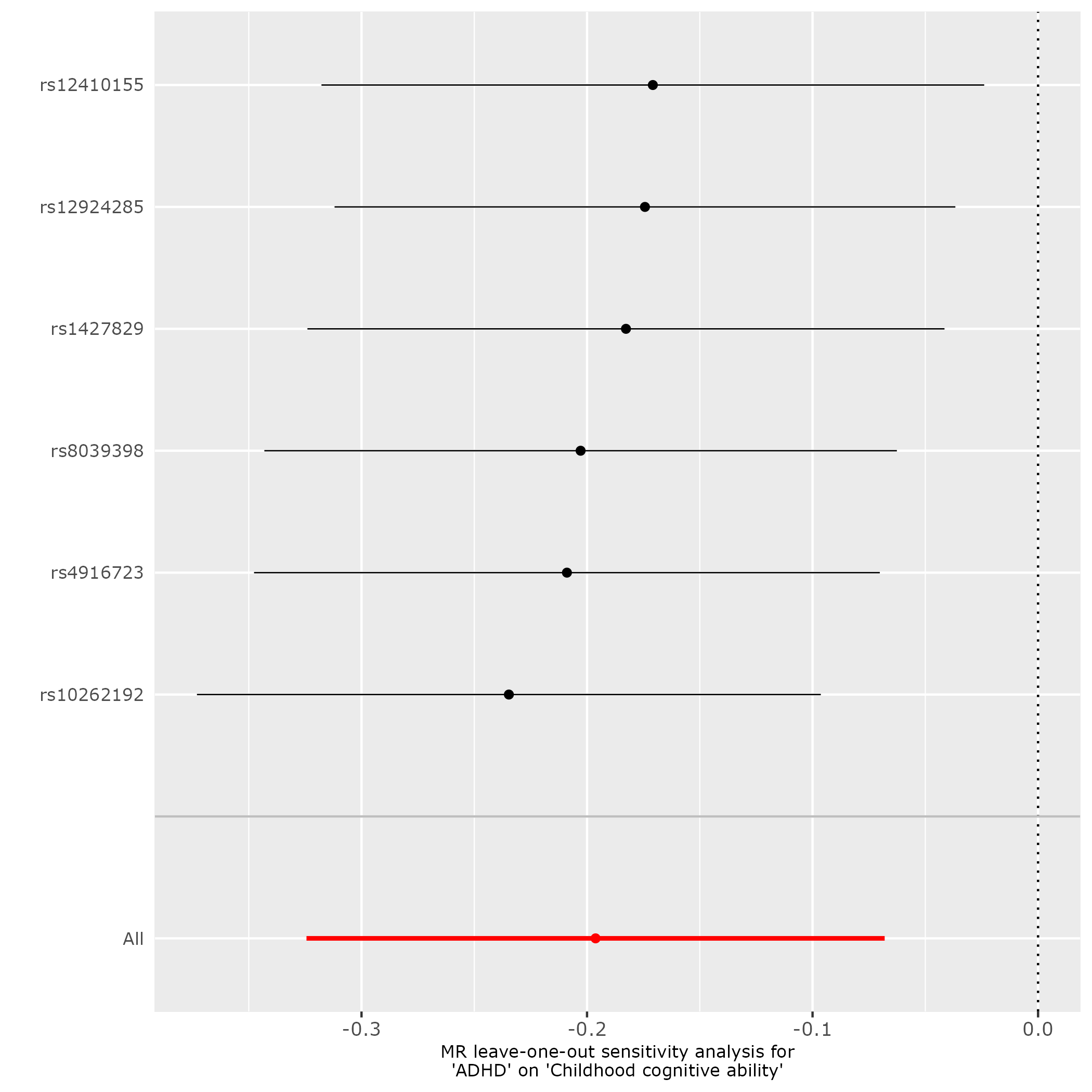


Supplementary Figure 3. Leave one out Analysis: ADHD🡪 Intelligence


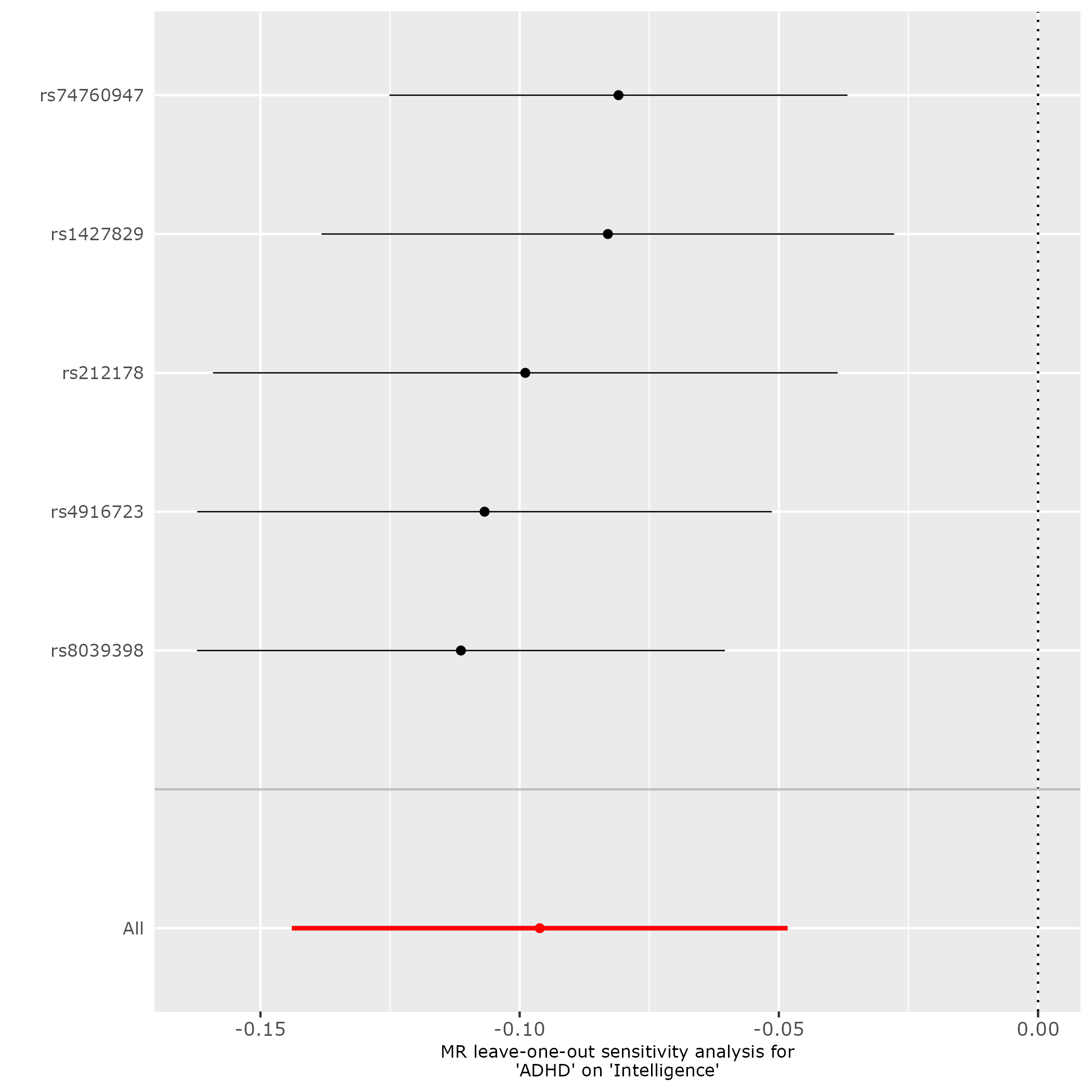


Supplementary Figure 4. Leave one out Analysis: ADHD🡪 Major Depression


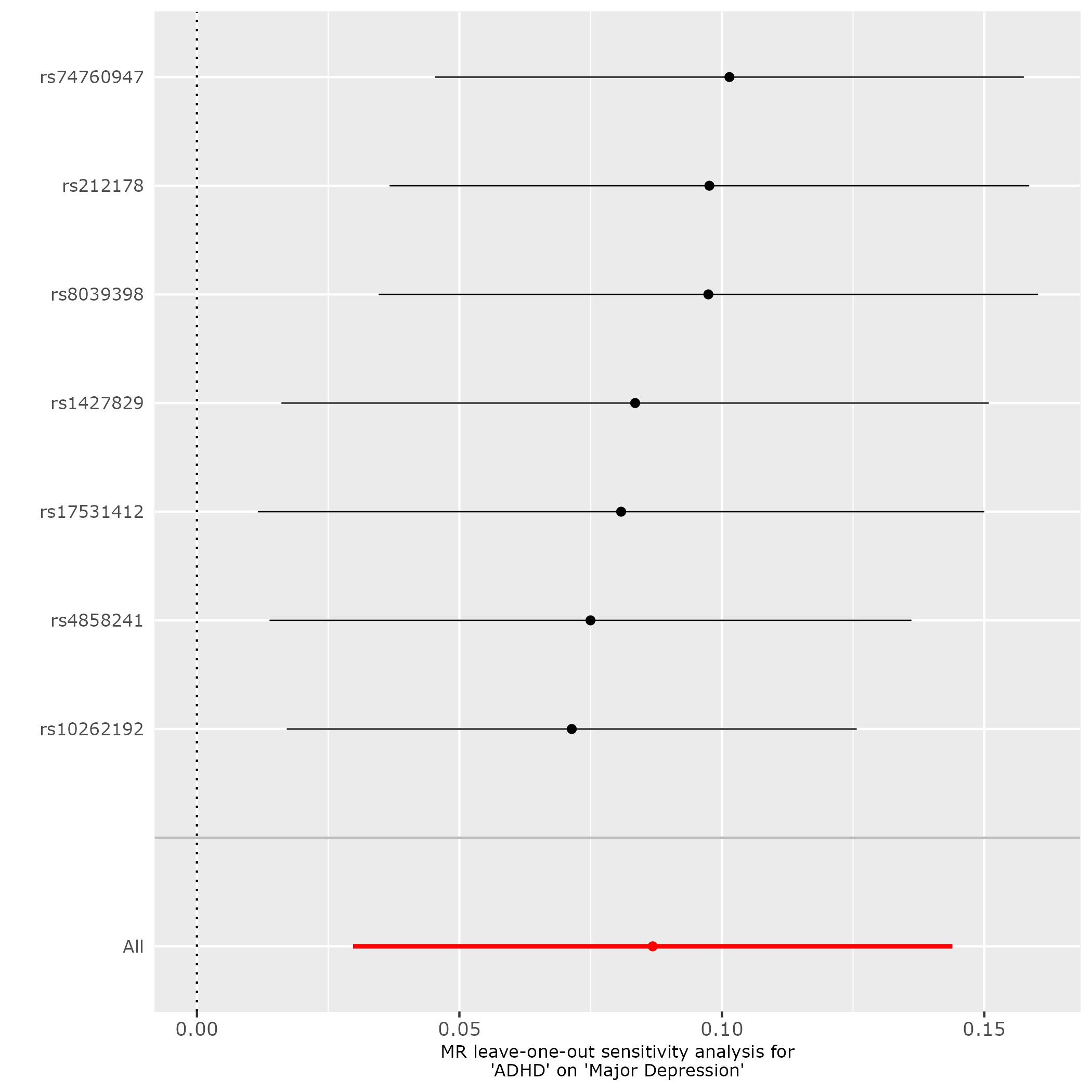


Supplementary Figure 5. Leave one out Analysis: ADHD🡪 ASD


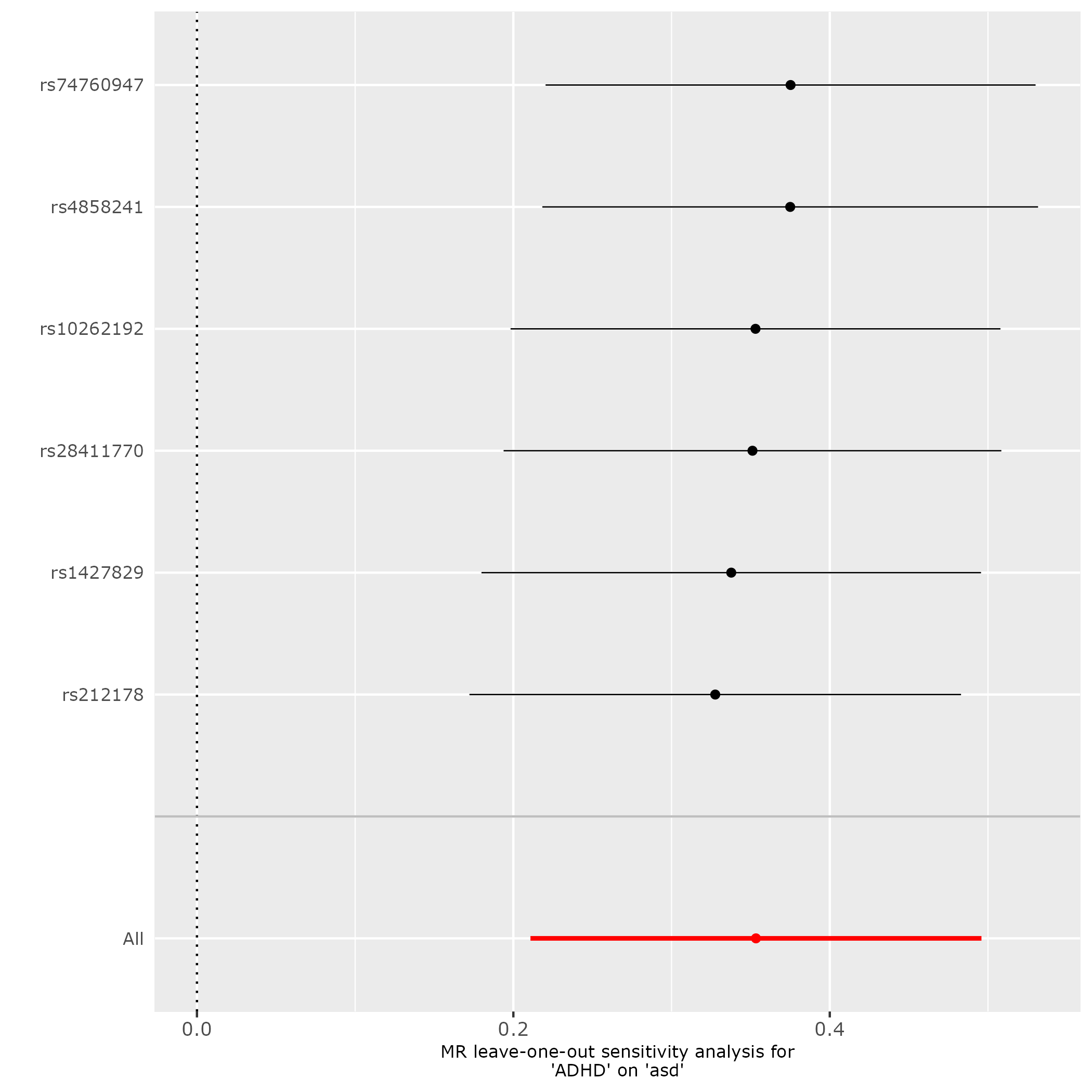


Supplementary Figure 6. Leave one out Analysis: Total Brain Volume 🡪 ADHD


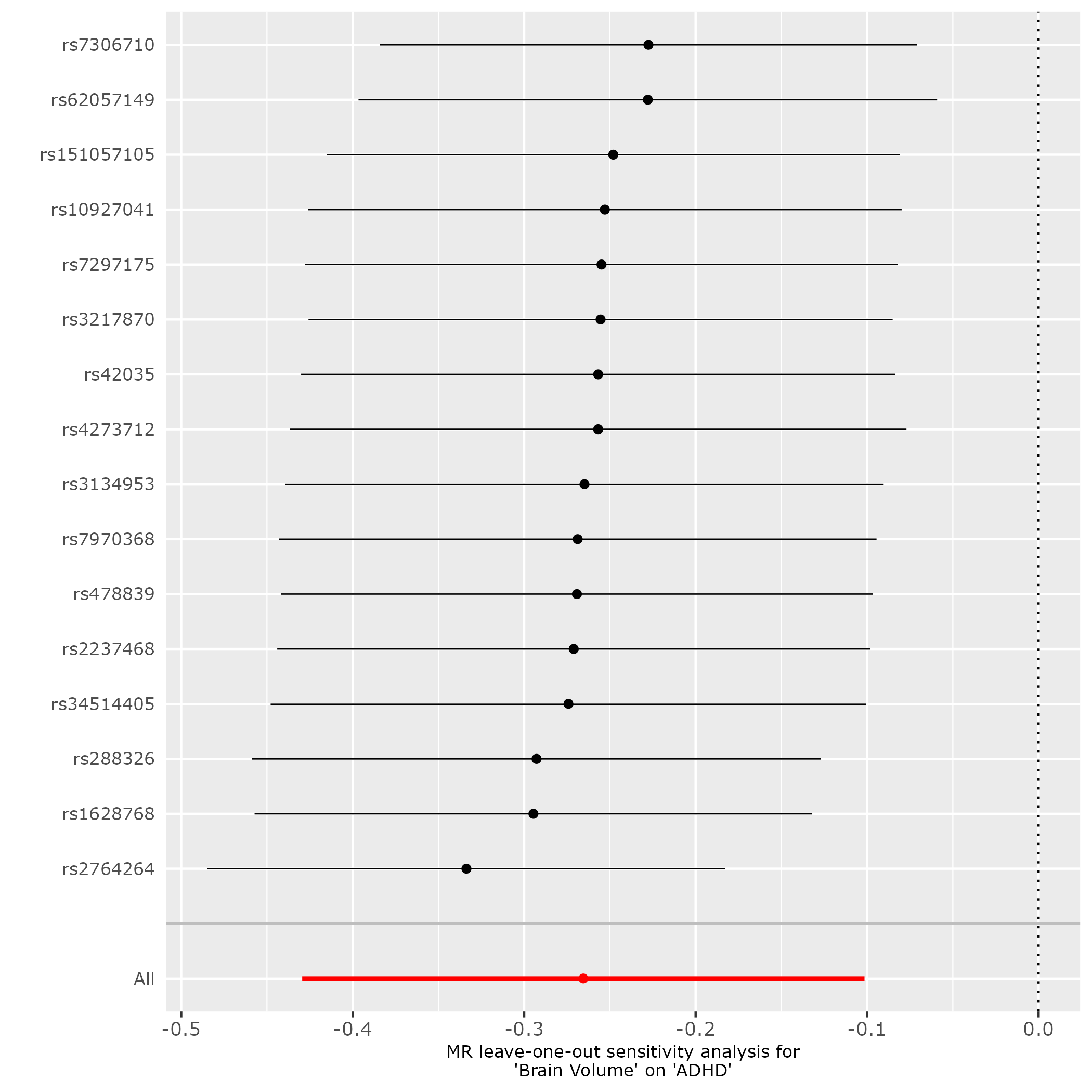


Supplementary Figure 7. Leave one out Analysis: Net Edge ICA2 🡪 ADHD


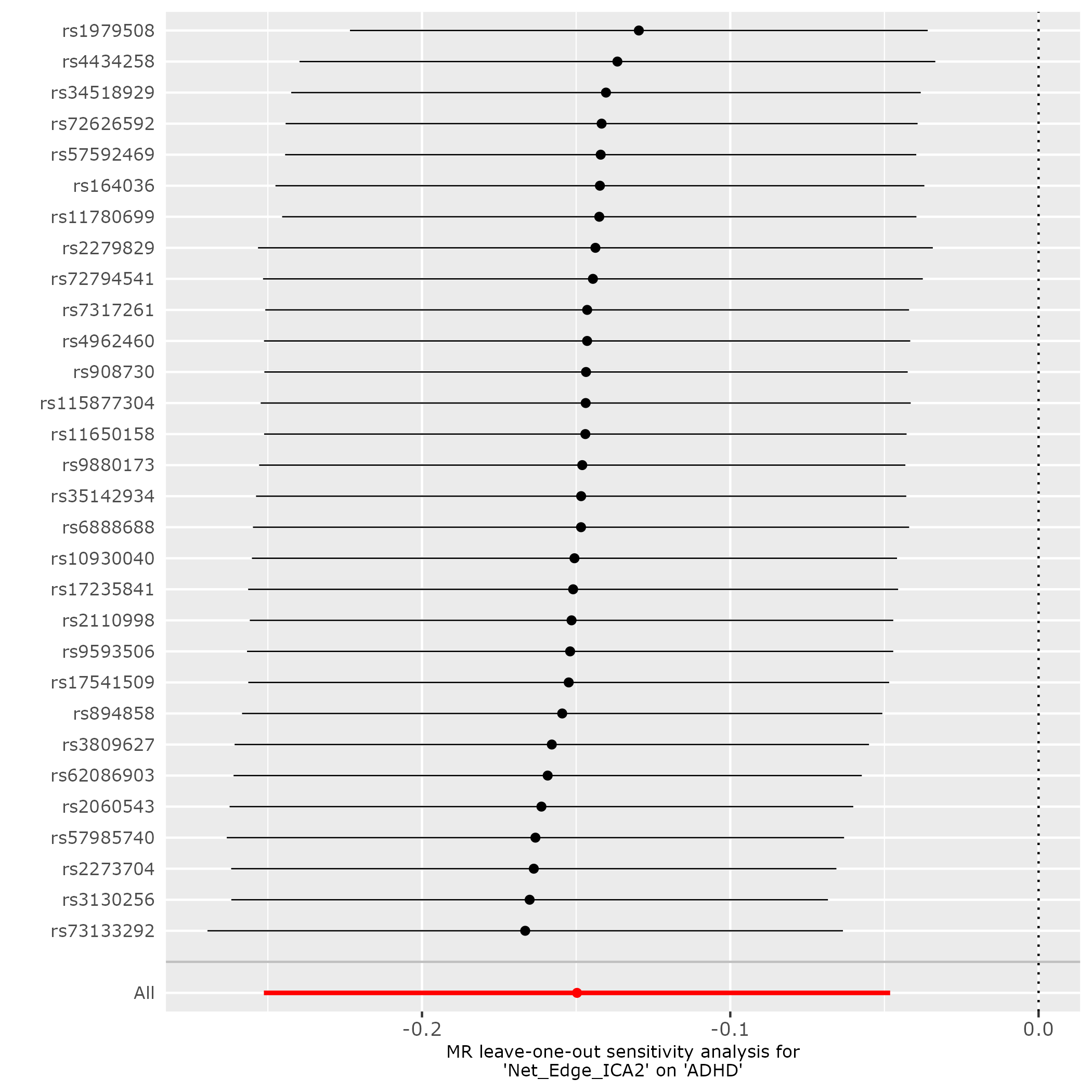


Supplementary Figure 8. Leave one out Analysis: Intelligence 🡪 ADHD


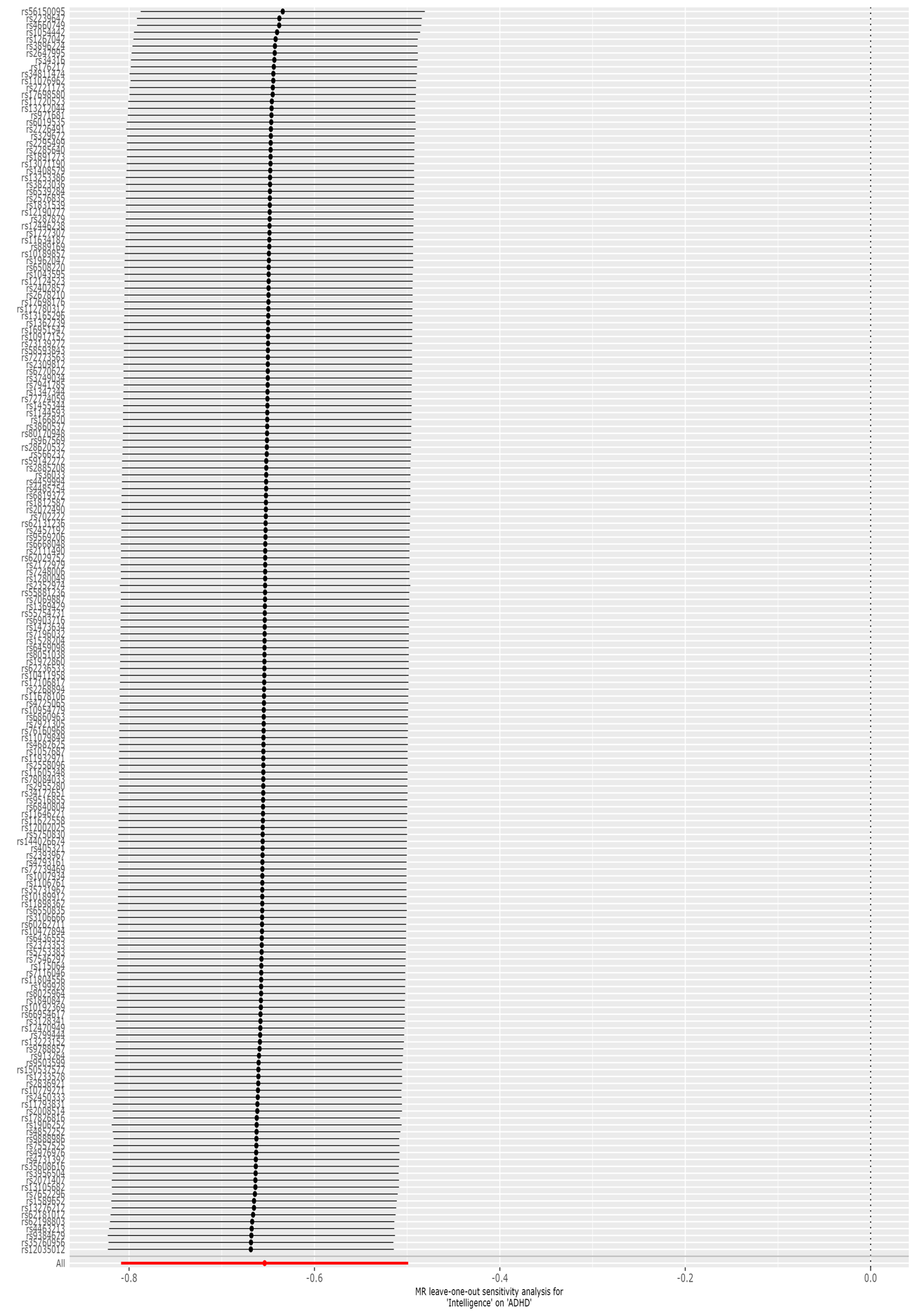


Supplementary Figure 9. Leave one out Analysis: Major Depression 🡪 ADHD


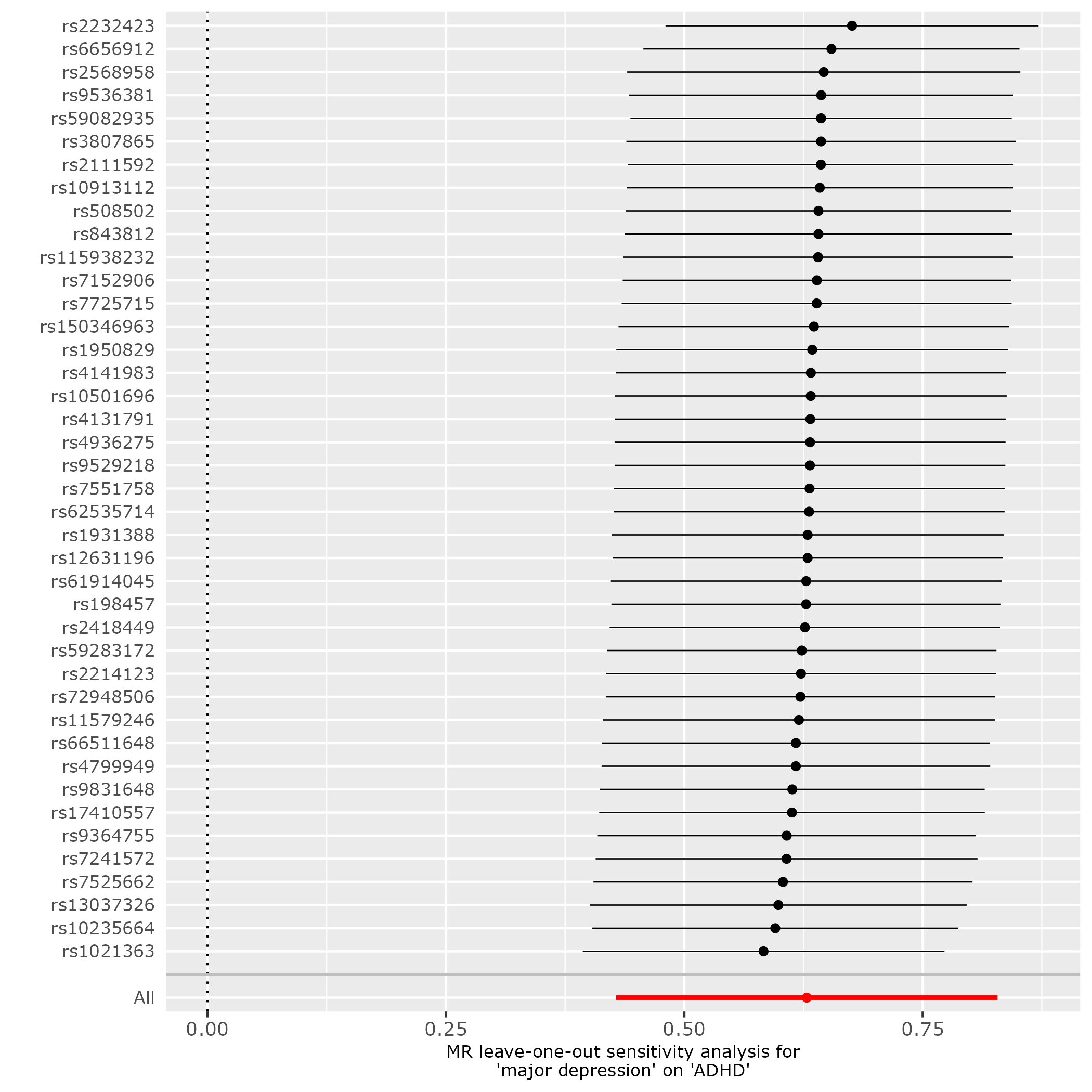


Supplementary Figure 10. Leave one out Analysis: ASD 🡪 ADHD


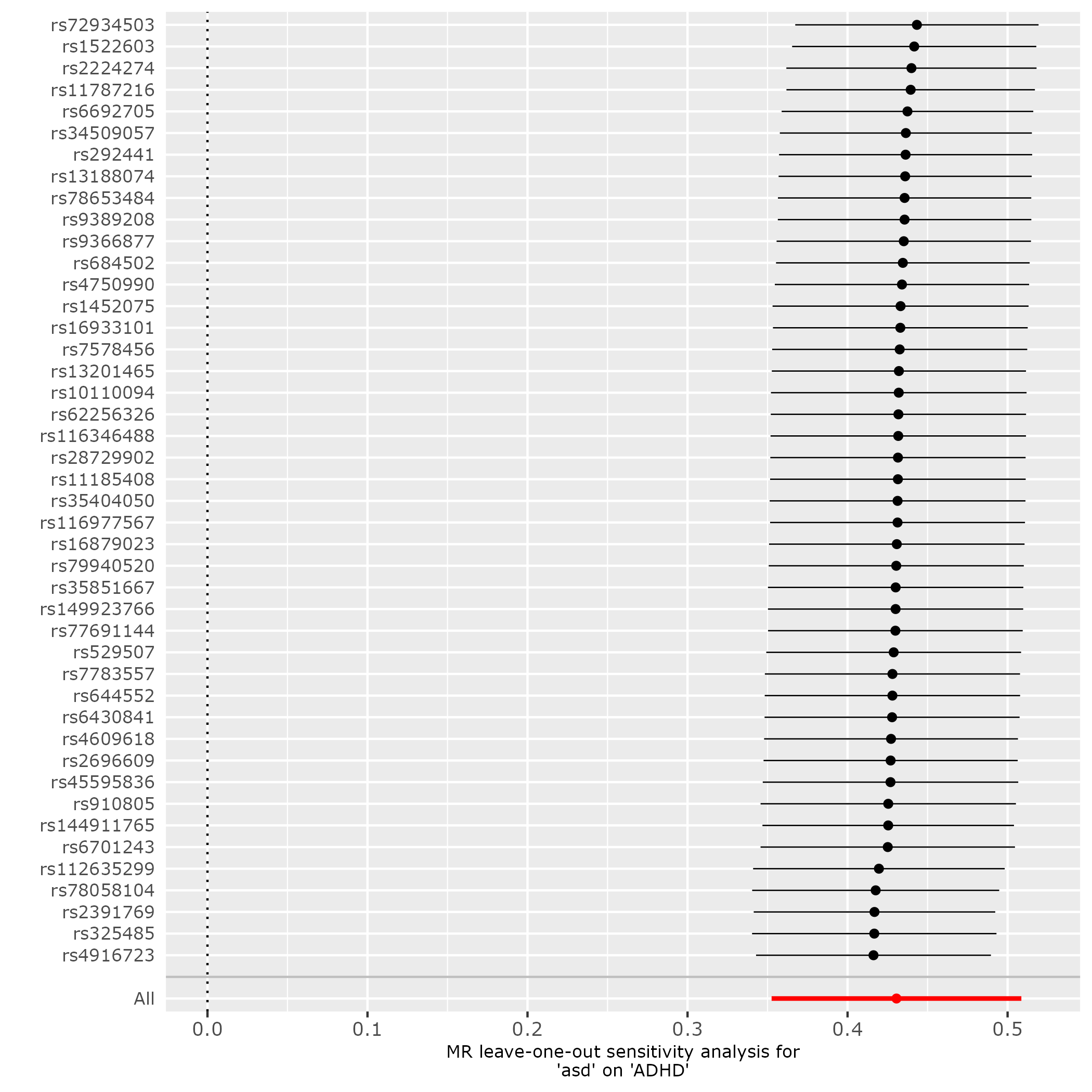

Supplement: Supplementary file 5 — Leave one out Analysis [file 41398_2022_2139_MOESM5_ESM.docx]
